# Effect of Ionic and Nonionic Compounds Structure on the Fluidity of Model Lipid Membranes: Computer Simulation and EPR Experiment

**DOI:** 10.3390/membranes14120257

**Published:** 2024-12-03

**Authors:** Dariusz Man, Barbara Pytel

**Affiliations:** Institute of Physics, Opole University, Oleska 48, 45-052 Opole, Poland

**Keywords:** liposomes, membrane rigidity, monte carlo simulation, EPR spectroscopy, spin probes

## Abstract

This article investigates the influence of dopant molecules on the structural and dynamic properties of lipid bilayers in liposomes, with a focus on the effects of dopant concentration, size, and introduced electric charge. Experimental studies were performed using electron paramagnetic resonance (EPR) spectroscopy with spin probes, complemented by Monte Carlo simulations. Liposomes, formed via lecithin sonication, were doped with compounds of varying concentrations and analyzed using EPR spectroscopy to assess changes in membrane rigidity. Parallel simulations modeled the membrane’s surface layer as a system of electric dipoles on a 20 × 20 rectangular matrix. As in the EPR experiments, the simulation explored the effects of dopant molecules differing in size and charge, while gradually increasing their concentrations in the system. Minimum binding energy configurations were determined from the simulations. The results revealed a strong correlation between the EPR data and simulation outcomes, indicating a clear dependence of membrane stiffening on the concentration, size, and charge of dopant molecules. This effect was most pronounced at low dopant concentrations (~1–1.5% for q = 2 and 1.5–2% for q ≥ 3). No significant stiffening was observed for neutral molecules lacking charge. These findings offer valuable insights into the mechanisms of membrane modulation by dopants and provide a quantitative framework for understanding their impact on lipid bilayer properties.

## 1. Introduction

Despite the vast diversity of life, cellular membranes share a remarkable structural consistency. All cells are enclosed by biological membranes, whose structures vary depending on their specific functions, but universally feature a lipid bilayer as the foundational element. This lipid bilayer serves as a matrix, incorporating proteins, carbohydrates, and other molecules that imbue the membrane with unique properties. Understanding the factors that influence lipid bilayer dynamics and structure is essential for unraveling the complex behaviors of biological membranes [1,2,3,4].

**Figure 1:**
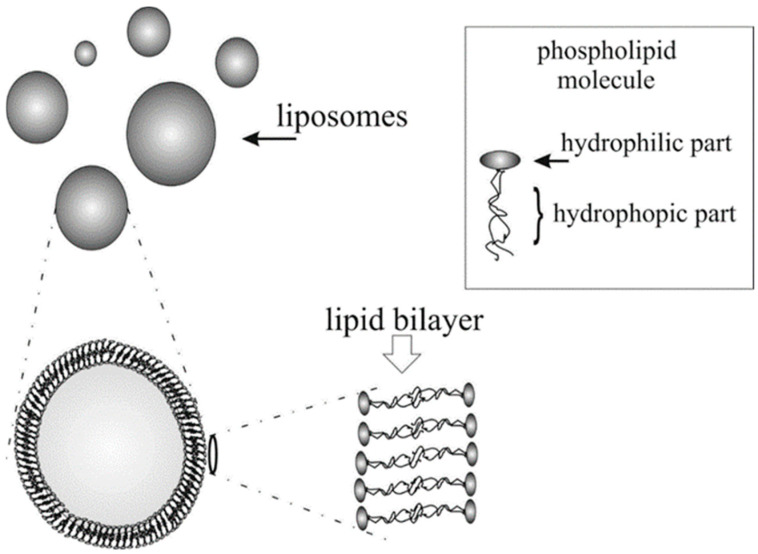
Structure of the lipid bilayer of monolayer liposomes formed in the lecithin sonication process.

Liposomes, which are artificial vesicles composed of lipid bilayers, serve as a robust model for studying the physical and chemical properties of biological membranes (Figure 1). By precisely controlling their molecular composition and size, liposomes allow researchers to explore membrane dynamics under well-defined yet sufficiently complex conditions, closely approximating natural membrane systems [5,6,7]. Additionally, liposomes have garnered significant interest in the pharmaceutical and cosmetic industries, particularly for their potential in drug delivery systems. The concept of using liposomes as “smart” carriers, capable of releasing therapeutic agents in a controlled manner at targeted locations, has spurred multidisciplinary research across physics, chemistry, biology, medicine, and computational sciences.

Targeted drug delivery using liposomes holds immense promise for enhancing therapeutic efficacy and minimizing side effects, particularly in oncology [8,9]. The encapsulation of chemotherapeutic agents within liposomes has already demonstrated clinical success in certain contexts [10]. Ongoing research continues to focus on developing more stable liposome formulations to ensure safe and efficient drug transport, a critical requirement for advancing this technology further [11,12,13,14,15].

However, despite the significant advances in liposomal drug delivery, studying biological membranes and their models remains a formidable challenge due to the membrane’s intrinsic complexity and heterogeneity. Early theoretical models [16,17,18,19] provided foundational insights into membrane behavior, focusing primarily on the hydrophobic core as a key determinant of membrane properties. With the advent of advanced computational techniques, the field has expanded to include a more comprehensive view of membranes as dynamic, multifaceted systems.

Computer simulations have become indispensable tools for investigating membrane behavior at the molecular level. Two primary approaches have emerged: deterministic methods, such as molecular dynamics (MD) simulations (e.g., GROMACS, CHARMM), which allow for time-dependent analysis of molecular interactions, and stochastic methods like Monte Carlo simulations, which introduce random sampling to model phenomena such as phase transitions. These approaches have shed light on processes such as phase separation in lipid bilayers and changes in binding energy at membrane interfaces [19,20,21].

Recent studies underscore the importance of electrostatic properties in membrane behavior. The dipolar nature of membrane headgroups and the influence of charged dopants have a significant impact on membrane fluidity and stability. In this study, we investigate how varying the concentration and charge density of ionic dopants affects the binding energy and fluidity of model lipid bilayers. Additionally, we examine the role of dopant size in modulating membrane properties, providing new insights into the complex forces governing biological membrane behavior.

In the experimental phase of this research, we explored the impact of ionic dopants with varying charges on the spectroscopic properties of liposome membranes. Liposomes were prepared by sonication of egg yolk lecithin (EYL). Spectroscopic analyses were conducted using electron paramagnetic resonance (EPR) with spin probes, a well-established technique for probing membrane dynamics. Changes in spin probe parameters were used to infer variations in membrane fluidity, enabling us to link dopant characteristics (charge, concentration) to modifications in membrane properties [22,23,24]. The use of chemically distinct spin probes, each localizing at different depths within the membrane, provided a cross-sectional view of membrane fluidity, enhancing our understanding of membrane heterogeneity.

## 2. Materials and Methods

### 2.1. Compounds

The dopants were chosen to represent a range of sizes and charges, consistent with those used in the computer simulations.

Small molecules:

Potassium chloride (KCl) and Tin(II)chloride(SnCl_2_). They were purchased at Sigma-Aldrich, St. Louis, MO, USA.

**Figure 2:**
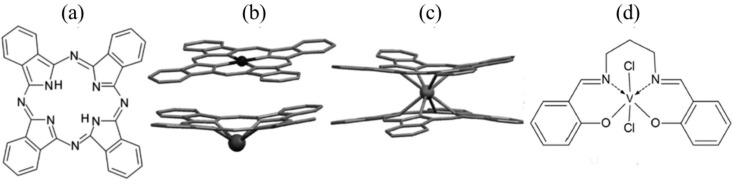
Chemical and molecular structures of phthalocyanine and its metal complexes. (**a**) Phthalocyanine (H_2_Pc), (**b**) SnPc, (**c**) NdPc_2_ (sandwich system), and (**d**) V-2propyl. The molecular structures of (**b**,**c**) are derived from the Cambridge Structural Database (*cif files: PTHCZN, SNPHCY01, CIZGIB02, and JUDNUR).

Large molecules:

Figure 2a—The phthalocyanine molecule (H_2_Pc)—Basic Structure it consists of four isoindole units (C_6_H_4_C_2_N) linked via nitrogen atoms, forming a 16-membered macrocyclic ring with alternating nitrogen and carbon atoms around the core. The original H_2_Pc structure has two hydrogen atoms in the center, which can be replaced with metal ion to create metallo-phthalocyanines (MPc).

N,N0-1,3-propylenebis (salicylideneiminate)—(V-2propyl) structural formula Figure 2d, Phthalocyanine SnPc and NdPc_2_, structural formula Figure 2b,c. These compounds were synthesized at the Faculty of Chemistry, University of Opole, Poland.

### 2.2. Spin Probes and Lecithin

Egg yolk lecithin (EYL) EYL (L-_-Lecithin, Azolectin, 3-sn-Phosphatidylcholine, PC, 1,2-Diacyl-sn-glycero-3-phosphocholine).

Spin probes used in EPR measurements for liposome labelling. TEMPO:

(2,2,6,6-tetramethylpiperidine-1-oxyl) and 16DOXYL: 2-ethyl-2-(15-methoxy-15-oxopentadecyl)-4,4-dimethyl-3-oxazolidinyloxy. Were purchased from Sigma-Aldrich.

### 2.3. EPR Measurements

**Figure 3:**
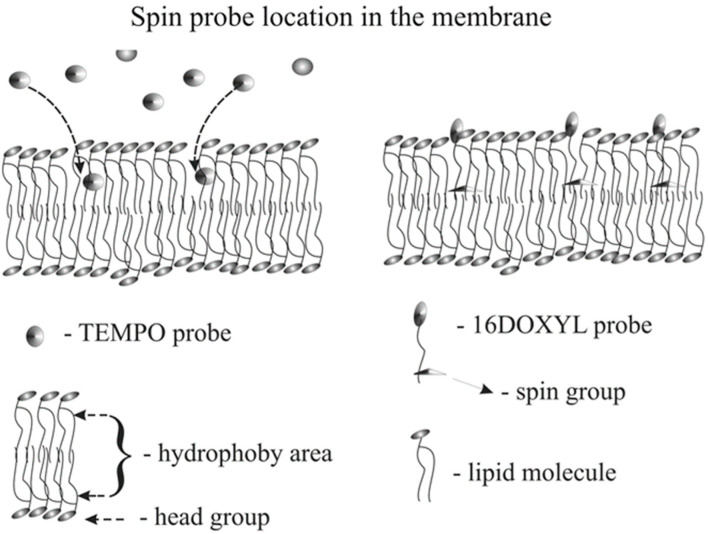
Schematic representation showing the localization of the spin probes in the lipid bilayer.

To assess the impact of ionic dopants on liposome membrane fluidity, electron paramagnetic resonance (EPR) spectroscopy was employed, using spin probes as molecular reporters of membrane dynamics. Two spin probes were selected: 16DOXYL-stearic acid methyl ester (16DOXYL), which localizes in the hydrophobic core of the membrane, and TEMPO, which resides closer to the membrane surface (Figure 3). These probes are well-known for their sensitivity to changes in membrane fluidity at distinct depths within the lipid bilayer.

Liposomes were prepared by sonication of egg yolk lecithin (EYL) in distilled water. A predetermined amount of lecithin solution in chloroform was transferred to a quartz vessel, and the solvent was allowed to evaporate completely under vacuum to ensure the formation of a thin film. Subsequently, distilled water was added to the vessel, and the mixture was subjected to sonication.

Sonication was performed using an ultrasonic disintegrator (UD-20; Techpan, Warsaw, Poland), with alternating cycles of 20 s of sonication and 30 s of cooling to prevent overheating, for a total duration of 5 min. As a result of this process, LUV liposomes are created, i.e., large single-layer liposomes.

Each sample contained 40 mM EYL lecithin in 1.5 mL of water. Following sonication, the resulting liposome suspension was divided into two parts, and spin probes (TEMPO or 16DOXYL) were added at a concentration of 500 ppm relative to lipid content. The samples were thoroughly mixed and allowed to equilibrate for 10 min to ensure uniform distribution of the spin probes within the lipid bilayer.

EPR measurements were performed at a constant temperature of 22 °C, and the total measurement time for each sample did not exceed 2 h. The EPR spectra were recorded using an MX-201R EPR spectrometer (TU Wroclaw, Poland) under the following operating conditions: microwave power p = 60 mW, sweep range ΔH = 7 mT, modulation amplitude dH = 0.08 mT, time constant dt = 0.1 s, and sweep time t = 128t s. Thin-walled glass capillaries were used to load the samples into the spectrometer cavity.

**Figure 4:**
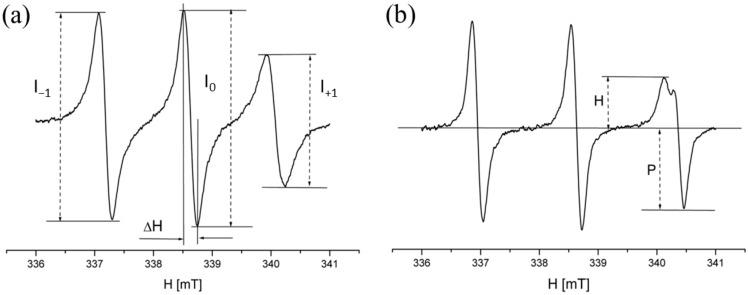
EPR spectra of spin probes placed in the liposome membrane. (**a**) 16DOXYLstearic acid methyl ester probe and rotation correlation time *τ = 5.95 · ΔH((I_*0*_/I_*+1*_)^*1/2*^ + (I_^0^_*/I*_*−1*_)^*1/2*^ − 2) 10 ^^−10^^ [s]*, (**b**) TEMPO probe and spectroscopic partition coefficient parameter *F = H/(H + P)*.

By monitoring changes in the spectral parameters of the spin probes, particularly hyperfine splitting and rotational correlation times, we evaluated the effects of dopants on membrane fluidity at different depths within the bilayer (Figure 4). The relative measurement errors were determined to be 3% for the spectroscopic partition coefficient parameter *F* and 4% for the rotational correlation time *τ*.

### 2.4. Computer-Based Model

To investigate the effects of ionic dopants on the surface layer of membranes, we conducted a series of computer simulations based on an extended version of a previously developed membrane surface model [22]. This model simulates the dipolar interactions between phospholipid headgroups, which are crucial for understanding membrane dynamics. For this study, the model was adapted to include algorithms for dopants with varying charge values, mobilities, and sizes. These dopants interact with the dipole matrix representing the polar headgroups of the lipid bilayer.

**Figure 5:**
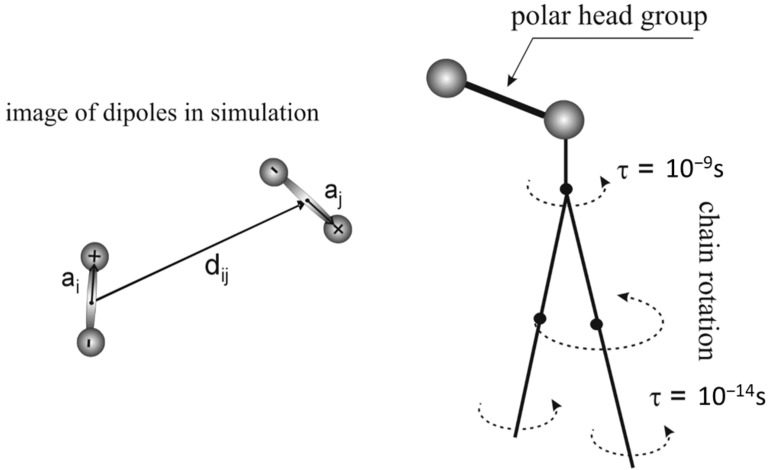
The parameterization of the dipoles and a schematic representation of the dynamics of phospholipid molecules.

The total energy of the system (Hamiltonian) is defined by:

$$H=\sum_{\left(i\right)}\frac{p_{i}^{2}}{2m}+\sum_{\left(i\right)}\frac{L_{i}^{2}}{2I}+\sum_{\left(i<j\right)}U_{ij}$$

where the first two terms represent the potential energy contributions from dipole-dipole interactions and dopant-dipole interactions. The interaction energy *U_ij_* between dipoles is governed by a combination of Coulomb’s law and Lennard-Jones potential:

$$U_{ij}=\frac{qiqj}{4\pi \varepsilon_{0}\varepsilon_{r}}\left(\frac{1}{\left|{\vec{d}}_{ij}+\vec{a_{j}}-\vec{a_{i}}\right|}-\frac{1}{\left|\vec{d_{ij}}-\vec{a_{j}}-{\vec{a}}_{i}\right|}+\frac{1}{\left|\vec{d_{ij}}-\vec{a_{j}}+\vec{a_{i}}\right|}-\frac{1}{\left|\vec{d_{ij}}+\vec{a_{j}}+\vec{a_{i}}\right|}\right)$$

$$4\varepsilon \left[{\left(\frac{\sigma }{\left|{\vec{d}}_{ij}\right|}\right)}^{12}-{\left(\frac{\sigma }{\left|{\vec{d}}_{ij}\right|}\right)}^{6}\right]$$

where qi and qj are the charges of the interacting particles, *d_ij_* is the distance between them, and *ϵ* and *σ* are the Lennard-Jones parameters. The mass of the dipole is denoted as m, and its moment of inertia is *I.* The parameters a_i_ and *a_j_* represent the distances from the dipoles’ axes of rotation (Figure 5).

The simulation employs the Metropolis Monte Carlo method, where each dipole undergoes random rotational (*dα*) and translational (*dx*) steps. These steps are governed by the following expression:

$$k=\frac{\pi }{l}\;\frac{\langle dx\rangle }{\langle d\alpha \rangle }$$

where *k* is a dimensionless parameter that modulates the average range of the dipoles’ translational and rotational movements. The system is iterated until it converges to a configuration of minimum total interaction energy, allowing an efficient exploration of the membrane’s energy landscape under different dopant conditions.

The proposed membrane model is relatively simple, focusing on Coulombic interactions: dipole-dipole, dipole-ion, and ion-ion interactions. It does not directly account for intermolecular interactions within the hydrophobic core of the membrane. As a result, the analysis is qualitative, as the model omits the direct influence of membrane lipid chains on the dynamics of dipoles moving across the membrane surface. In this study, these interactions are assumed to be stochastic and may impact different degrees of freedom to varying extents.

In specific scenarios, lipid chains can limit the complete displacement of the dipole axis, stabilizing the membrane and causing it to oscillate around an average value. The configuration of states in this system is determined by the parameter *k* and the system’s temperature. The simulation concludes when the system reaches the minimum binding energy, which represents the state in which the elements of the system are most strongly bound to each other, achieving maximum stability under the given boundary conditions. In the context of membranes, this state can be interpreted as a reduction in membrane fluidity—essentially, a stiffening of the membrane under these specific conditions.

## 3. Results and Discussion

### 3.1. Simulation Results

The primary aim of the simulations was to explore the behavior of the surface layer of the membrane in the liquid crystal phase at 295 K under the influence of increasing concentrations of ionic impurities, varying their charge and size. Dipoles were arranged in a rectangular 20 × 20 matrix, allowing free rotation around their symmetry axis and movement within the XY plane. Initially, dipole orientations and impurity distributions were randomized using a number generator. Sufficient simulation steps were conducted to enable the system to reach equilibrium, defined as the minimum binding energy.

**Figure 6:**
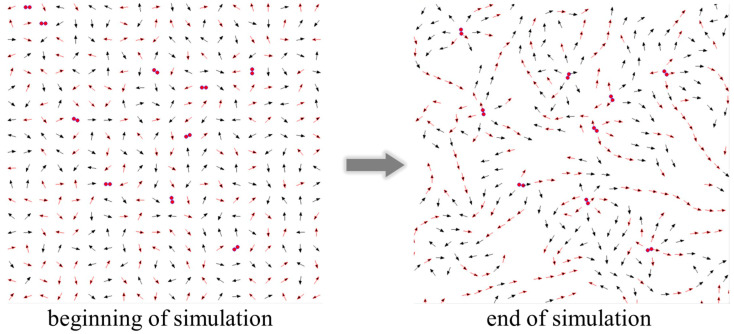
Examples of 20 × 20 bitmaps of dipoles with dopants of size 0.3 nm and charge 2q = 2. The simulation began with random placements, concluding when the system reached minimum energy.

The dipole dimensions were set at l = 0.5 nm, consistent with values reported by Stigter et al. (1992) [25] for phosphatidylcholine. The initial spacing between dipole centers was set to 2l [26]. Alongside binding energy, the simulation captured bitmap images at each step, enhancing visualization of the system’s evolution (Figure 6).

**Figure 7:**
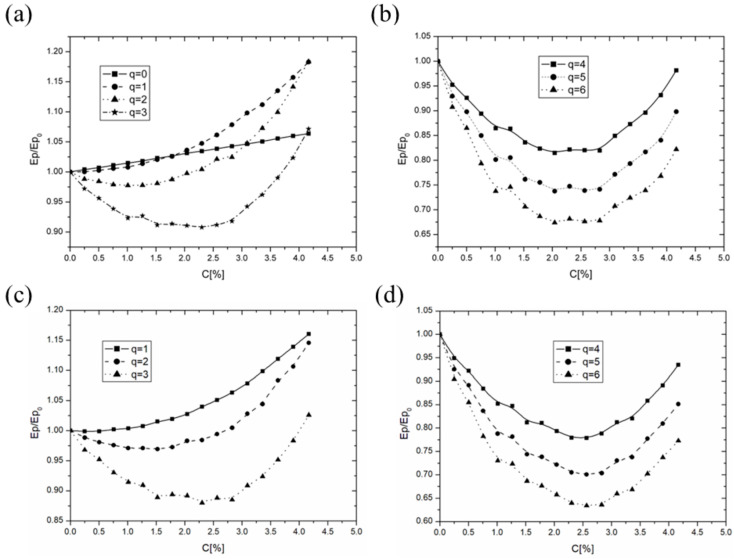
Relative binding energy Ep/Ep_0_ as a function of dopant concentration C [%]: (**a**,**b**) for a dopant size of L = 0.1 nm with varying charge q; (**c**,**d**) for a dopant size of L = 0.5 nm with varying charge q.

Figure 7 illustrates the relationship between the binding energy of the dipole system and the concentration, size, and charge of ionic dopants. A range of dopant sizes from L = 0.1 nm to 0.7 nm was examined, capturing a wide spectrum of potential molecules within the membrane. Given the extensive data generated, only representative examples are presented to illustrate the observed trends. To emphasize changes due to dopants, the energy values were normalized by dividing the current binding energy Ep by the initial binding energy Ep_0_ (i.e., Ep/Ep_0_).

Graphs a and b (Figure 7) depict the binding energy dependence on ionic dopant concentration for a size of L = 0.1 nm. The data indicate that initially, as doping begins, the system’s energy decreases until a minimum is reached, after which it starts to rise. The concentration at which this minimum occurs varies with impurity charge: for q = 1, this effect is minimal; for q = 2q, the minimum occurs at approximately 1.2–1.5%; and for q = 3, 4, 5 and 6, the minimum concentration ranges from about 2% to 3%. In contrast, uncharged impurities (q = 0) exhibited a linear relationship without any observable extremum.

Similar trends were noted for dopants sized at L = 0.5 nm (Figure 7c,d), with more pronounced extremes and lower minimum energy values compared to the L = 0.1 nm dopants. An increase in system energy can be interpreted as a reduction in membrane binding forces, leading to increased membrane fluidity. The results indicate that the incorporation of uncharged dopants progressively diminishes binding forces, potentially resulting in complete membrane disintegration. Conversely, charged molecules initially reduce membrane fluidity, but exceeding a certain concentration reverses this effect, increasing fluidity.

**Figure 8:**
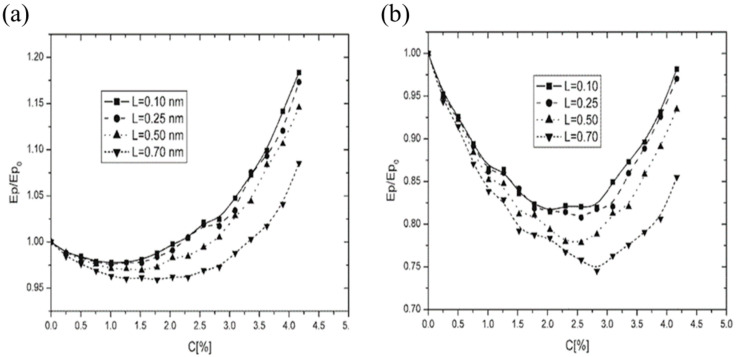
Relative binding energy Ep/Ep0 as a function of dopant concentration C [%] for dopants ranging from L = 0.1 nm to L = 0.7 nm: (**a**) charge q = 2, (**b**) charge q = 4.

The influence of dopant size on the depth of the energy minimum is illustrated in Figure 8. The data indicate that larger dopants exert a greater influence on the energy minimum’s depth. The shallowest minimum was associated with L = 0.1 nm, while the most pronounced minimum occurred for L = 0.5 nm. Additionally, the charge value of the impurity significantly affects the depth of the extremum, as demonstrated in Figure 8a (charge q = 2) and Figure 8b (chargé q = 4).

The dependence of the binding energy of the system on the concentration of ionic impurities, as illustrated in the graphs in Figure 7 and Figure 8, can be explained as follows: Initially, as ionic impurities are introduced to the dipole system, there is a decrease in the binding energy. This decrease reaches a minimum at a specific impurity concentration, creating a parabolic shape in the energy curve. This behavior can be attributed to Coulomb interactions: introducing charges into the dipole matrix enhances Coulombic forces between dipoles and impurities, resulting in a stronger, more stable system with lower binding energy. However, as impurity concentration continues to increase beyond this critical point, repulsive forces between similarly charged impurities become more pronounced. These repulsive interactions begin to outweigh the stabilizing dipole-charge interactions, leading to an increase in binding energy and a reduction in system stability. This trend suggests a delicate balance where ionic impurities initially stabilize the system through attractive forces, but excessive concentrations disrupt it due to repulsion. The location and depth of the energy minimum are influenced by both the charge and size of the impurities, as larger or more highly charged impurities tend to shift the minimum and alter the stability profile of the system.

Interestingly, the effect of impurity size on the depth of the minimum binding energy is unexpected. One might intuitively assume that larger impurities would disrupt the structure, thereby reducing binding forces and “flattening” the minimum. However, our observations indicated the opposite effect: larger ionic impurities actually further reduced the system’s binding energy, thereby enhancing its stability. This phenomenon could have significant implications for understanding real membrane behavior in the presence of ionic impurities, suggesting that impurity size plays a more complex role in membrane stabilization than previously anticipated. These findings highlight a close relationship among concentration, size, and charge of the impurities and membrane fluidity, particularly the presence of a minimum in binding energy correlating with maximum membrane stiffness.

### 3.2. EPR Experiment

To investigate the influence of dopants on liposome membrane fluidity, EPR spectroscopy was employed using spin probes. The selected dopants represented a diverse spectrum of properties in terms of size and electric charge (Figure 4).

#### 3.2.1. Rotational Correlation Time Analysis

**Figure 9:**
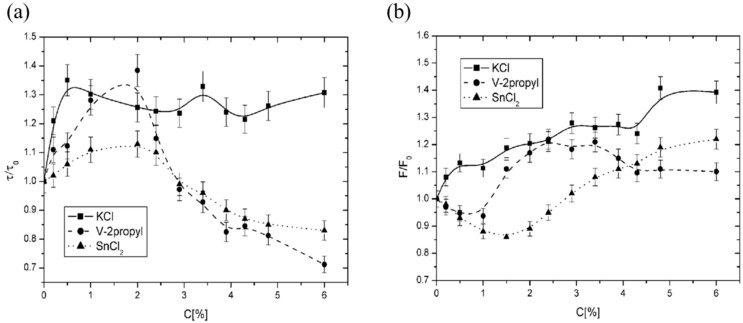
(**a**) Effect of dopants on the central region of the liposome membrane as assessed by EPR measurements with the 16DOXYL spin label. *τ/τ_0_* represents the relative rotational correlation time parameter; values < 1 indicate increased fluidity, while values > 1 indicate decreased fluidity. (**b**) Effect of dopants on the water-lipid interface as assessed by EPR measurements with the TEMPO spin label. F/F0 represents the relative partition parameter; values > 1 indicate increased fluidity, and <1 indicate decreased fluidity.

Figure 9a displays the dependence of the rotational correlation time parameter (*τ*) of the 16DOXYL probe on dopant concentration. An increase in τ/*τ_*0*_* indicates a slowing of probe rotation, suggesting enhanced membrane stiffness [27]. The rotational correlation time can be calculated using the formula:

$$\tau =5.95\;\cdot \;△Ho\cdot \left(\;\sqrt{\frac{I_{0}}{I_{+1}\;\;}}+\sqrt{\frac{I_{0}}{I_{-1}}}-2\;\right)\cdot 10^{-10}\;[s]$$

where *I*_0_, *I*_−1_, and *I*_+1_ are parameters detailed in Figure 4a. To emphasize the changes caused by dopants, the τ values were normalized by dividing the current values by the initial value, *τ*_0_ (*τ/τ*_0_). The results show that during the initial phase of doping, the *τ/τ*_0_ ratio increases, reaching maximum values for KCl around 0.5%, and for SnCl_2_ and V-2propyl in the range of 1.5% to 2%. The increase in this parameter indicates a slowdown in the probe’s rotation, as its rotational time around the symmetry axis increases. This phenomenon corresponds to an increase in the viscosity of the membrane environment where the probe is located, implying a decrease in membrane fluidity. After reaching these maxima, the *τ/τ*_0_ ratio decreases significantly for SnCl_2_ and V-2propyl, while only minor changes are observed for KCl. A reduction in the τ/τ_0_ ratio corresponds to faster probe rotation, indicating a decrease in viscosity and thus an increase in membrane fluidity.

**Figure 10:**
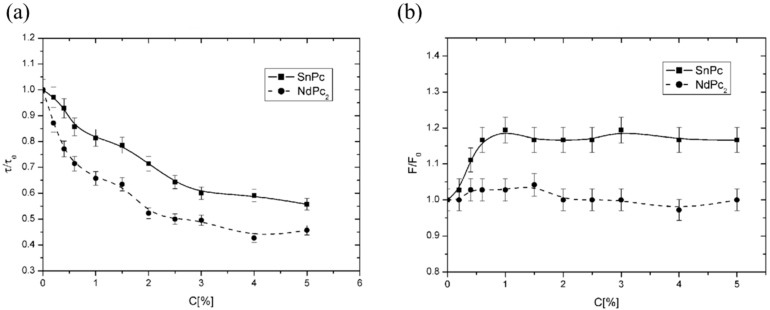
(**a**) Effect of dopants on the central region of the liposome membrane as assessed by EPR measurements with the 16DOXYL spin label. τ/τ_0_ represents the relative rotational correlation time parameter; values < 1 indicate increased fluidity, while values > 1 indicate decreased fluidity. (**b**) Effect of dopants on the water-lipid interface as assessed by EPR measurements with the TEMPO spin label. *F/F_0_* represents the relative partition parameter; values > 1 indicate increased fluidity, while values < 1 indicate decreased fluidity.

Figure 10a shows the dependence of τ of the 16DOXYL probe on dopant concentration. As in previous analyses, *τ* values were normalized to 1. The results indicate that as dopant concentration increases, *τ* decreases, signifying increased membrane fluidity. Above a concentration of 2.5%, a plateau is observed, suggesting the maximum solubility of the tested compounds in the membrane was reached. Notably, the maximum change in τ due to NdPc_2_ was approximately 55%, while changes due to SnPc were around 45%, indicating a stronger effect of NdPc_2_ on membrane fluidity.

#### 3.2.2. Partition Coefficient Analysis

Figure 9b illustrates the dependence of the partition coefficient (*F*) parameter of the TEMPO probe on dopant concentration. The TEMPO probe used in the experiment, due to its amphiphilic properties, can incorporate into both polar and non-polar environments (Figure 3). This property is leveraged to monitor changes in the fluidity of liposome membranes [28]. When the probe is introduced into a dispersion of liposomes suspended in water, it integrates into both the aqueous and lipid phases, resulting in a splitting of the high-field line into two distinct lines. This phenomenon arises from the differing effects of polar and non-polar environments on the probe. Under fixed thermodynamic conditions, the ratio of these two lines remains constant, enabling the determination of the spectroscopic parameter *F* (partition parameter). This parameter provides insight into the distribution of the probe between the two phases, reflecting the probe’s environment and thereby serving as an indirect measure of membrane fluidity. The *H* line represents the probe incorporated into the lipid environment, while the *P* line indicates the probe residing in the aqueous environment. Changes in the ratio between these two lines reflect the migration of the probe between the aqueous and lipid phases. A decrease in *F* indicates the displacement of the probe from the lipid membrane towards the aqueous phase, suggesting increased membrane stiffness. The partition coefficient can be expressed as:

$$F=\frac{H}{\left(H+P\right)}\;$$

where *H* and *P* are defined in Figure 4b. As before, the values of *F* were normalized to highlight changes, resulting in a ratio *F/F_0_*. The data reveal that during initial doping, F decreases, with minimum values observed for V-2propyl around 0.75% and for SnCl2 around 1.5%; no minimum was observed for KCl. Following these minima, *F* gradually increased with rising dopant concentrations. This increase indicates enhanced solubility of the probe in the liposome bilayer, reflecting greater membrane fluidity.

Figure 10b presents the dependence of the partition parameter *F* of the TEMPO probe on dopant concentration. An increase in *F* suggests heightened solubility of the probe in the membrane, indicative of increased fluidity. Normalization was performed similarly as in previous cases. The data show that as dopant concentrations rise, *F* increases for SnPc, indicating membrane fluidification. In contrast, for NdPc2, changes remained within the margin of error, implying a negligible effect on the outer layer fluidity. Thus, SnPc had a more substantial impact on membrane surface characteristics, likely due to its hydrophobic nature enabling deeper integration into the membrane’s hydrophobic core.

It can also be observed that the SnPc compound, at concentrations above 1%, does not induce further changes in the *F* parameter. This suggests the existence of a limiting concentration for SnPc in the surface layer of the membrane. Beyond this threshold, the molecules likely shift from the surface layer into the hydrophobic interior of the membrane.

## 4. Conclusions

This study aimed to evaluate the proposed membrane model’s effectiveness in elucidating the processes occurring within lipid bilayers under the influence of various compounds. The findings confirmed the presence of a maximum membrane stiffness associated with compounds that contribute an electric charge (Figure 9a,b). A tendency was also observed for the extreme value to shift towards higher concentrations as the charge of the dopant increased (Figure 7a,c). Conversely, compounds that did not introduce any charge into the membrane did not exhibit a clear extremum in fluidity, which aligns with the predictions of the model (Figure 7a, q = 0).

Additionally, it was demonstrated that the size of the dopants influenced both the position of the extremum on the concentration axis and the magnitude of the changes in fluidity (Figure 8a,b—computational model; Figure 9a,b—EPR experiment).

The presented computer model of the membrane surface layer simplifies the complex interactions that occur in real membranes, omitting many of the subtle molecular interactions. Nonetheless, it highlights the significant role of Coulomb interactions in determining the behavior of the lipid bilayer when exposed to charged compounds. Coulomb interactions appear to be a decisive factor in the doping of real and model membranes with compounds that introduce uncompensated electric charges. Changes in liposome membrane fluidity due to dopants, as a function of concentration, have been observed and reported in numerous studies [22,29,30,31,32]. Such research, ongoing for several decades, remains crucial to the development of techniques utilizing liposomes, as evidenced by more recent studies [33,34].

These findings are particularly significant for the design of liposomes for drug delivery, a field that continues to evolve despite persistent challenges related to membrane stability [7,35,36,37,38]. Moreover, the proposed model contributes to the understanding of the dynamics of natural biological membranes, particularly cell membranes, and the stabilizing role that various dopants can play. By integrating experimental EPR data with computational simulations, this study provides a comprehensive view of the effects of ionic dopants on membrane properties. The spectroscopic changes observed in the spin probes illustrate how dopants of varying charges and concentrations impact membrane fluidity, while the simulations elucidate the underlying energetic interactions between dopants and membrane dipoles. This interdisciplinary approach fosters a deeper understanding of how charged molecules influence lipid bilayer stability and dynamics, thereby contributing to the broader knowledge of membrane functionality in both biological and model systems.

## Data Availability

Data is contained within the article.
